# The potential effects and mechanisms of breast inflammatory lesions on the occurrence and development of breast cancer

**DOI:** 10.3389/fonc.2022.932743

**Published:** 2022-08-05

**Authors:** Zhaoxia Chang, Ying Zhang, Jue Fan, Lixing Zhang, Suling Liu, Guangyu Liu, Juchuanli Tu

**Affiliations:** ^1^ Fudan University Shanghai Cancer Center and Institutes of Biomedical Sciences, State Key Laboratory of Genetic Engineering, Cancer Institutes, Key Laboratory of Breast Cancer in Shanghai, The Shanghai Key Laboratory of Medical Epigenetics, Shanghai Key Laboratory of Radiation Oncology, The International Co-laboratory of Medical Epigenetics and Metabolism, Ministry of Science and Technology, Shanghai Medical College, Fudan University, Shanghai, China; ^2^ Department of Breast Surgery, Fudan University Shanghai Cancer Center, Shanghai, China; ^3^ Department of Oncology, Shanghai Medical College, Fudan University, Shanghai, China; ^4^ Singleron Biotechnologies, Nanjing, China

**Keywords:** breast tumor, inflammation-cancer transformation, inflammatory molecules, transcriptomics, early diagnosis

## Abstract

Breast cancer as the most common cancer in women has become the leading cause of cancer death for women. Although many inflammatory factors increase the risk of breast cancer, there are very few studies on the mechanisms by which inflammation affects the initiation and progression of breast cancer. Here, we profiled and compared the transcriptome of normal tissues, inflammatory breast tissues, benign breast tumors, and malignant breast tumors. To find key regulatory factors, a protein interaction network between characteristic modules in inflammatory lesions and ER-negative (ER^−^) breast cancer was constructed and inflammation-cancer interface genes were identified. We found that the transcriptional profile of inflammatory breast tissues was similar with ER^−^ malignant tumors, featured with low ER expression levels and similar immune signaling pathway activation. Through comprehensive protein network analysis, we identified the interface genes and chemokine signaling pathway that have the potential to promote inflammatory cancer transformation. These interface genes could be used as a risk factor to provide a certain basis for the clinical early detection and treatment of breast cancer. This is the first study to explore the association between breast inflammatory lesions and breast cancer at the transcriptome level. Our inflammation data and research results provide a basis for future inflammation-cancer transformation analysis.

## Introduction

Breast cancer as the most common cancer in women has become the leading cause of cancer death for women (1). Because of its high heterogeneity in molecules and phenotypes, breast cancer is traditionally divided into four clinical subtypes: Luminal A, Luminal B, HER2-positive (Her2^+^), and triple-negative breast cancer (TNBC), according to the expression of estrogen receptor (ER), progesterone receptor (PR), and human epidermal growth factor receptor 2 (HER2) (2, 3). On the basis of the characteristics of the transcription profile, breast cancer can also be divided into five “intrinsic” molecular subtypes: LumA, LumB, Her2^+^, basal and normal-like, and the concordance between molecular subtypes and clinical subtypes can reach about 70%–80% (4, 5). Although many factors that increase the risk of breast cancer have been reported, the exact carcinogenic mechanism of breast cancer is still unknown (6, 7). Because of the many similarities between inflammation and cancer, people have been working on the relationship between them for many years (8). Since 1863, the inflammation-cancer transformation model was proposed, that is, cancer occurs from chronic inflammation, more and more studies have proved that inflammation can lead to the occurrence and development of cancer (9, 10). Although there is some evidence of the carcinogenic effects of inflammation in many cancer types, such as hepatocellular carcinoma (HCC) and colorectal cancer (CRC), the link between breast cancer and inflammation is still poorly understood (11, 12). Chronic inflammation exists in breast cancer and makes an important contribution to the infiltration of lymphocytes and macrophages (12, 13). Therefore, it is pointed out that chronic inflammation in the breast may have a certain effect on the occurrence of breast cancer. Recently, a retrospective study of patients in Taiwan found that patients with mastitis will have a higher risk to developed into breast cancer in the future (14), implying the possibility of inflammatory lesions transforming into cancer.

Here, we profiled and compared the expression profiles of normal, inflammatory, benign tumor, and malignant tumor tissues. To analyze the relationship between inflammatory lesions and breast cancer more comprehensively, we included four subtypes of invasive breast cancer: Luminal A, luminal B, Her2^+^, and TNBC. By comparing the expression profiles of characteristic genes and the activation of signal pathways, we found that the inflammatory lesions of the breast were more similar to the two ER^−^ breast cancer subtypes: Her2^+^ breast cancer and TNBC. Using network analysis to integrate the expression profile of inflammation and ER^−^ breast cancer including Her2^+^ breast cancer and TNBC, we identified the genes at the interface between inflammation and cancer modules. From these interface genes, we speculate that chemokine signaling pathway and genes may be key factors for inflammatory cancer transformation, and some of these genes can be used as prognostic factors.

## Materials and methods

### Samples collections

All breast tissue samples from 46 cases were collected from patients admitted to Fudan University Cancer Hospital. An informed consent was obtained for all patients, and the study was approved by the institution’s ethics committee (Fudan University Shanghai Cancer Center Institutional Review Board, 050432-4-1212B) (Shanghai, China). The patients were examined by professional clinicians, and the biopsy sample for each patient was checked by professional pathologists. The phenotype classification of samples was identified based on stained subtype-specific molecular markers by these professional pathologists. According to the phenotype classification, the samples fell into five categories: five normal breast tissues (NB), five inflammatory breast tissues (IBT), five fibroadenoma (Fibro) samples, five ductal carcinoma samples *in situ* (DCIS), and 26 invasive ductal carcinoma (IDC) samples. On the basis of receptor molecular status, 26 IDC samples were further divided: five Luminal A, 11 Luminal B, five Her2^+^, and five TNBC samples.

### Library preparation and sequencing

Total RNA was isolated and purified using TRIzol reagent (Invitrogen, Carlsbad, CA, USA) following the manufacturer’s procedure. The RNA amount and purity of each sample was quantified using NanoDrop ND-1000 (NanoDrop, Wilmington, DE, USA). The RNA integrity was assessed by Bioanalyzer 2100 (Agilent, CA, USA) with RIN number >7.0 and confirmed by electrophoresis with denaturing agarose gel. Poly(A) RNA was purified from 1 μg of total RNA using Dynabeads Oligo (dT)25-61005 (Thermo Fisher, CA, USA) using two rounds of purification. Then, the poly(A) RNA was fragmented into small pieces using Magnesium RNA Fragmentation Module (NEB, cat. e6150, USA) under 94°C 5–7 min. Then, the cleaved RNA fragments were reverse-transcribed to create the cDNA by SuperScript™ II Reverse Transcriptase (Invitrogen, cat. 1896649, USA), which were next used to synthesize U-labeled second-stranded DNAs with *E. coli* DNA polymerase I (NEB, cat. m0209, USA), RNase H (NEB, cat.m0297, USA), and Deoxyuridine Triphosphat (dUTP) solution (Thermo Fisher, cat. R0133, USA). An A-base is then added to the blunt ends of each strand, preparing them for ligation to the indexed adapters. Each adapter contains a T-base overhang for ligating the adapter to the A-tailed fragmented DNA. Single- or dual-index adapters are ligated to the fragments, and size selection was performed with AMPureXP beads. After the heat-labile UDG enzyme (NEB, cat. m0280, USA) treatment of the U-labeled second-stranded DNAs, the ligated products are amplified with PCR by the following conditions: initial denaturation at 95°C for 3 min; eight cycles of denaturation at 98°C for 15 s, annealing at 60°C for 15 s, and extension at 72°C for 30 s; and then final extension at 72°C for 5 min. The average insert size for the final cDNA library was 300 ± 50 bp. At last, we performed the 2×150-bp paired-end sequencing (PE150) on an illumina Novaseq™ 6000 (LC-Bio Technology CO., Ltd., Hangzhou, China) following the vendor’s recommended protocol.

### Read mapping and gene expression summary

High-quality reads were aligned to the human hg19 reference genome by STAR (15) version 2.6, and the number of reads on each gene was counted by featureCounts (16) version 2.0.0. The expression levels for each gene was normalized to fragments per kilobase of exon model per million mapped fragments by DESeq2 (17).

### Identification of differential expressed genes

DESeq2 (17) was used for differential expression analysis in our dataset. Limma (18) was used for differential expression analyses for RNA sequencing (RNA-seq) and microarray data in GEO database (GSE162694, GSE4183, and GSE83687). The final screening criterion is that absolute value foldchange is greater than 1.5, and the adjusted p-value is less than 0.05.

### Identification of dynamic change modules

We identified the differentially expressed genes between different subtypes of breast disease relative to normal samples (IBT *vs*. NB, ER^−^ *vs*. NB, Her2^+^ *vs*. NB, and TNBC *vs*. NB). We performed k-means cluster analysis based on the expression profiles of these differentially expressed genes and finally obtained 13 gene expression clusters.

### Negative-positive network construction

To construct negative-positive (NP) network, we extracted protein–protein interactions with confidence score of PPIs > 900 from HPRD (19), STRING database (20), and Wu’s parsed protein interaction network (21). We constructed a protein–protein interaction network between the IBT_Her2^+^_TNBC module and the Her2^+^_TNBC module. In the network, we only retained the interactions with the absolute value of the correlation coefficient between gene expression greater than 0.22. In the NP network, we defined genes that were bounded to genes in a different module as interface genes.

### Estimation of immune cell and erythroid cell abundance

The LM22 signature CIBERSORT algorithm (22) was used to estimate the proportion of immune infiltration of different immune cell types in each sample. The LM22 signature includes 547 genes, which can distinguish 22 types of immune cells. The correlation coefficient between genes and immune cell characteristics in TCGA data was obtained through GEPIA2 (23). To assess the erythroid cell infiltration, we obtained signature genes from published single-cell sequencing data (24). In this study, the erythroid cells were further divided into seven subtypes that were mature_RBCs, Transition_differentiating cells, ACVR2B_type, F cells, Reticulocytes, HEMGN_type, and NIX_type, respectively. We analyzed the infiltration of these seven subtypes. Using CIBERSORT algorithm (22) to predict the overall infiltration level of erythroid cells based on the signature genes for these seven subtypes.

### Functional enrichment analysis

The functional enrichment analysis of annotated terms from Gene Oncology (GO) and Kyoto Encyclopedia of Genes and Genomes (KEGG) were performed with online tool DAVID (25) and clusterProfiler (26).

### Gene set variation analysis

Gene set variation analysis (GSVA) and graphing were done by R package “GSVA” (27).

### Statistical analysis

Comparisons of the ratio of immune cells between two groups were performed by Student’s t-test. The correlation between gene expression and samples was measured by Pearson correlation coefficient. Chi-square test was used to test the correlation between gene sets.

### Survival analysis

Cox regression analysis was used to detect the influence of gene expression value on survival rate. Cox regression and Kmplot visualization used survival package and survminer package (28, 29). The expression profile and clinical data of breast cancer were downloaded from the TCGA database. We divided the samples into high expression groups and low expression groups according to the median value of gene expression risk scores.

### Compliance and ethics

All breast tissue samples from 46 cases were collected from patients admitted to the Fudan University Cancer Hospital. An informed consent was obtained for all patients, and the study was approved by the institution’s ethics committee (Fudan University Shanghai Cancer Center Institutional Review Board, 050432-4-1212B) (Shanghai, China). No potential conflicts of interest need to be disclosed by the listed authors.

## Results

### Inflammatory breast tissues and ER^−^ breast tumors shared similar transcriptional profile

**Figure 1:**
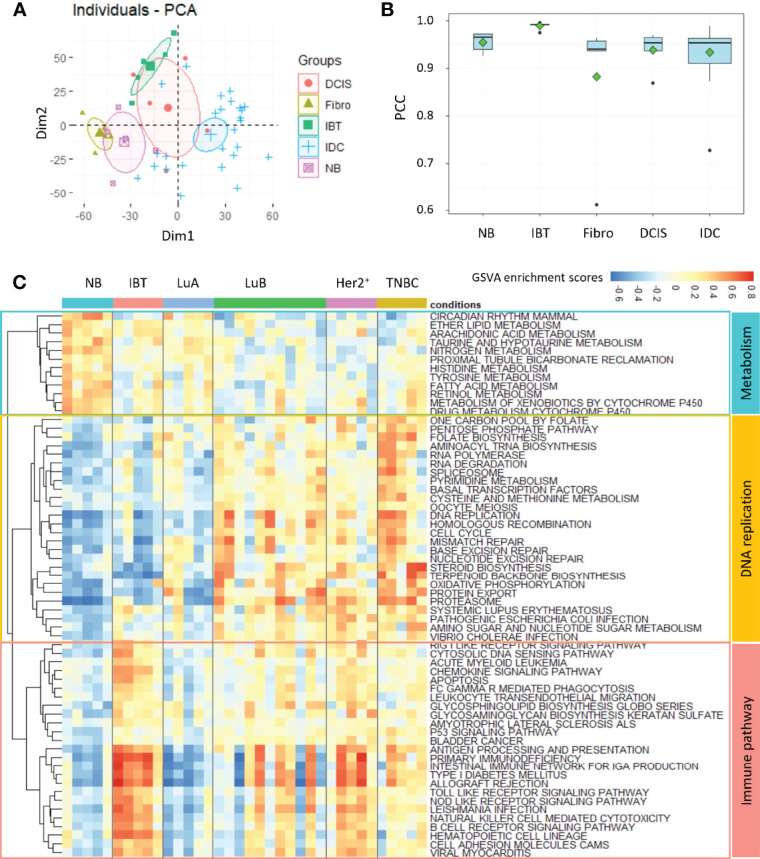
Inflammatory breast tissues and ER^−^ breast tumors shared similar transcriptional profile. **(A)** PCA analysis of gene expression profile among different breast tissues. NB, normal breast tissue; IBT, inflammatory breast tissue; Fibro, fibroadenoma; DCIS, ductal carcinoma *in situ*; IDC, invasive ductal carcinoma. **(B)** The Pearson correlation coefficient (PCC) of each sample’s transcriptome with the average gene expression profiles of all genes in IBT samples. **(C)** Heatmap of differentially activated KEGG pathways in NB, IBT, and IDC. IDC: Luminal A (LuA), Luminal B (LuB), Her2^+^, and TNBC.

To explore the relationship between inflammatory breast tissues and breast tumors, we collected a total of 46 breast tissue samples from patients admitted to the Fudan University Cancer Hospital for transcriptome profiling by next-generation sequencing technologies. The patients were examined by professional clinicians, and the biopsy sample for each patient was checked by professional pathologists. The phenotype classification of samples was identified based on stained subtype-specific molecular markers by these professional pathologists. We carefully checked the clinical data for these patients, especially for the five patients with inflammatory lesion and found that none of these patients was in suckling period. The samples fell into five categories: five normal breasts (NB), five inflammatory breast tissues (IBT), five fibroadenoma (Fibro), and five Ductal carcinomas *in situ* (DCIS), and 26 invasive ductal carcinoma (IDC) samples. The detailed clinical information for these patients was listed in **Supplementary Table 1**. On the basis of receptor molecular status, 26 IDC samples were further divided: five Luminal A, 11 Luminal B, five Her2^+^, and five TNBC subtype samples. Principal component analysis of the expression profile of 46 samples showed that the normal breast tissues (NB) and benign tumors (Fibro) were clearly distinguishable from malignant tumors (DCIS and IDC) (**Figure 1A**). By calculating correlation coefficients between expression of the genes in each sample and the average expressions of genes in IBT-type samples, we found that the similarity between IBT and malignant tumors was higher than that of fibroadenoma (**Figure 1B**). To further identify the similarity between IBT and malignant tumors, we used the GSVA method to estimate variation of signaling pathway activity in NB, IBT, and invasive breast cancer samples. Several metabolic pathways were specifically enriched in normal samples, whereas replication-related signaling pathways were mainly activated in invasive breast cancer. As expected, immune-related signaling pathways including B cell receptor signaling pathway and chemokine signaling pathway were activated in IBT, and we found that these pathways were also activated in IDC (**Figure 1C**). In particular, in patients with Her2^+^ breast cancer and TNBC, the proportion of immune-related signal pathway activation was higher. PAM50 is a 50-gene signature that is now commonly employed to identify breast cancer intrinsic subtypes (4, 5). Therefore, we tested the expression of the characteristic gene PAM50 in IBT to explore the similarity of intrinsic gene expression between IBT and invasive breast cancer. As displayed in **Supplementary Figure 1**, two of the five IBT patients were classified as Basal-like and three of the five IBT patients were classified as normal-like. Furthermore, we also detected the mRNA expression of ER, PR, and Her2 (ERBB2) in IBT and invasive breast cancer (**Supplementary Figure 1**). We found that the receptor transcription levels of different types of breast cancer diseases were basically consistent with the results of IHC. Compared with normal tissues, there was no statistically significant change in the expression levels of PR and Her2 in IBT. Interestingly, the expression of ESR1 in IBT was significantly downregulated, which was consistent with the expression changes of Her2^+^ breast cancer and TNBC samples. Because the expression level of ER in Her2^+^ breast cancer and TNBC are both negative, we collectively merged and named Her2^+^ breast cancer and TNBC as ER^−^ breast cancer. On the basis of expression of characteristic genes and activation of signaling pathways, we found that the transcriptome of IBT was more similar to ER^−^ breast cancer than other types of breast cancer.

### Immune response–related pathways were specifically enriched in IBT_Her2^+^_TNBC module

**Figure 2:**
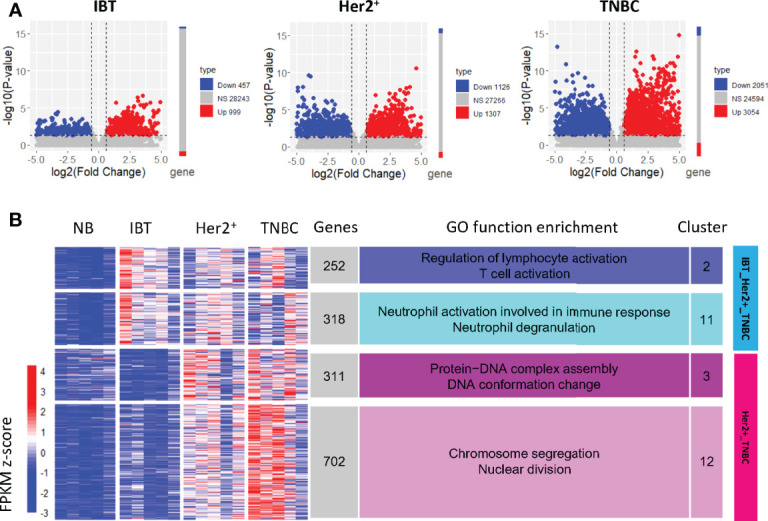
Gene clusters expressed in normal, inflammation, and ER^−^ breast cancer. **(A)** Differentially expressed genes in each breast diseases samples compared with NB samples. Compared with normal samples, genes with upregulated expression are represented in red, genes with downregulated expression are represented in blue, and NS represents other genes. **(B)** The expression clusters of differentially expressed genes in IBT and ER-negative samples (Her2^+^ and TNBC). The main enriched functional items are listed on the right. The heatmap on the left showed the normalized expression value of the differentially expressed genes.

To better explore the possible conversion relationship between IBT and ER^−^ breast cancer (Her2^+^ and TNBC), we identified the differentially expressed genes of different types of breast disease relative to normal samples (IBT *vs*. NB, ER^−^ *vs*. NB, Her2^+^ *vs*. NB, and TNBC *vs*. NB). The number of differentially expressed genes in ER^−^ breast cancer was significantly more than that in IBT, suggesting that the transcriptional dysregulation of genes in ER^−^ breast cancer was more pronounced than in IBT (**Figure 2A**). To detect the potential dynamic changing modules of the transition from inflammation to cancer, we performed k-means cluster analysis based on the expression profiles of these differentially expressed genes and finally obtained 13 gene expression clusters (**Supplementary Figure 2**). We normalized the expression values of all genes and used the average expression of cluster genes as the expression value of each cluster. The expression trends of clusters showed that most gene clusters were specifically and highly expressed in a certain type of breast disease. Interestingly, genes in cluster2 and cluster11 were highly expressed in IBT, Her2^+^ breast cancer, and TNBC samples; thus, we defined these two clusters as an IBT_Her2^+^_TNBC module (**Figure 2B**). Because genes in cluster3 and cluster12 were obviously highly expressed in Her2^+^ and TNBC breast cancer, they were defined as a Her2^+^_TNBC module (**Figure 2B**). Gene ontology (GO) analysis showed that genes in the IBT_Her2^+^_TNBC module were related with neutrophil activation involved in immune response, neutrophil degranulation, regulation of lymphocyte activation, and T cell activation. The genes in the Her2^+^_TNBC module were mainly enriched in chromosome segregation, nuclear division, protein–DNA complex assembly, and DNA conformation change.

### Chemokines and chemokine receptors were enriched in inflammation-cancer interface

**Figure 3:**
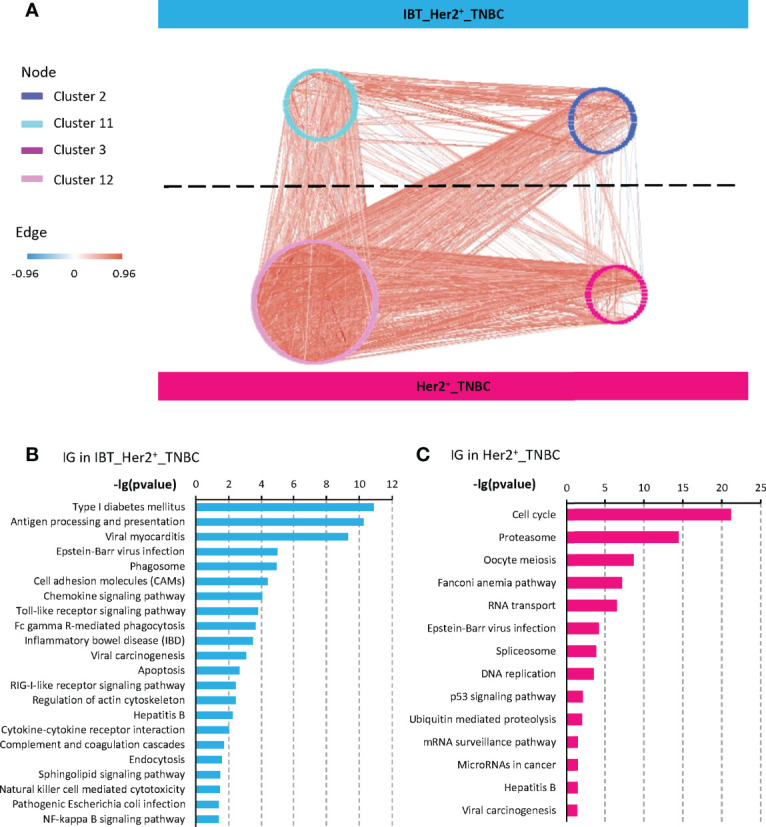
Interface genes were detected by protein interaction network. **(A)** A NP network generated between the four gene clusters from IBT_Her2^+^_TNBC module (clusters 2 and 11) and Her2^+^_TNBC module (clusters 3 and 12). Nodes represent genes, red edges represent positively correlated interactions between genes, and blue edges represent negatively correlated interactions. **(B, C)** KEGG terms enriched in interface genes (IG) from IBT_Her2^+^_TNBC module and Her2^+^_TNBC module.

Studies have shown that chronic inflammation can provide a beneficial immune microenvironment for early cancer. Therefore, those immune genes and pathways that highly expressed in tumors and related with cell proliferation must be key for inflammation-cancer transformation. Previous studies have found that the expression-based protein–protein interaction network between feature modules in different states could enrich key regulatory factors, which can drive state changes in the network (30, 31). To find these key genes involved in the transformation from inflammatory lesions to cancer, we constructed a positive-negative–correlated protein–protein interaction network (NP network) between the IBT_Her2^+^_TNBC module and the Her2^+^_TNBC module. We pointed out that the interactions between the IBT_Her2^+^_TNBC module (highly expressed in IBT and ER negative breast cancer) and the Her2^+^_TNBC module (high-expressed in ER^−^ breast cancer) can establish a link between IBT and ER^−^ breast cancer, so we named it “inflammation-cancer interface”. In the NP network, we define genes that are bounded to genes in a different module as interface genes (**Figure 3A**). Finally, 133 and 278 interface genes were identified in the IBT_Her2^+^_TNBC module and Her2^+^_TNBC module, respectively.

To identify the signaling pathways that play key roles in the transformation from inflammatory to cancer, we performed a KEGG signaling pathway enrichment analysis on interface genes. Interface genes in the IBT_Her2^+^_TNBC module were mainly related to immune signaling pathways, among which most enriched signaling pathway is the chemokine signaling pathway (**Figure 3B**). Multiple chemokines and chemokine receptors were found in interface genes (CCR1, CCR5, CXCL5, CXCL9, and CXCR4 in IBT_Her2^+^_TNBC module, and CXCL10 and CXCL11 in Her2^+^_TNBC module). As the known cancer driving factors, chemokines can recruit specific cytokines that promote cancer progression to trigger the tumor initiation site and form inflammatory microenvironment at the initial stage of cancer (10, 32, 33). At the same time, we also found that the signaling pathways related to enteritis and hepatitis, which were classic inflammation-cancer–transformed diseases, are also activated. It implied that breast inflammation might increase the risk of transformation to cancer. The interface genes in the Her2^+^_TNBC module were mainly enriched in cell cycle and cancer-related signaling pathways, such as the P53 signaling pathway (**Figure 3B**). It indicated that the interface genes in the Her2^+^_TNBC module may be related to cell expansion in early cancer.

### Interface genes had a prognostic role in inflammation-cancer transformation

**Figure 4:**
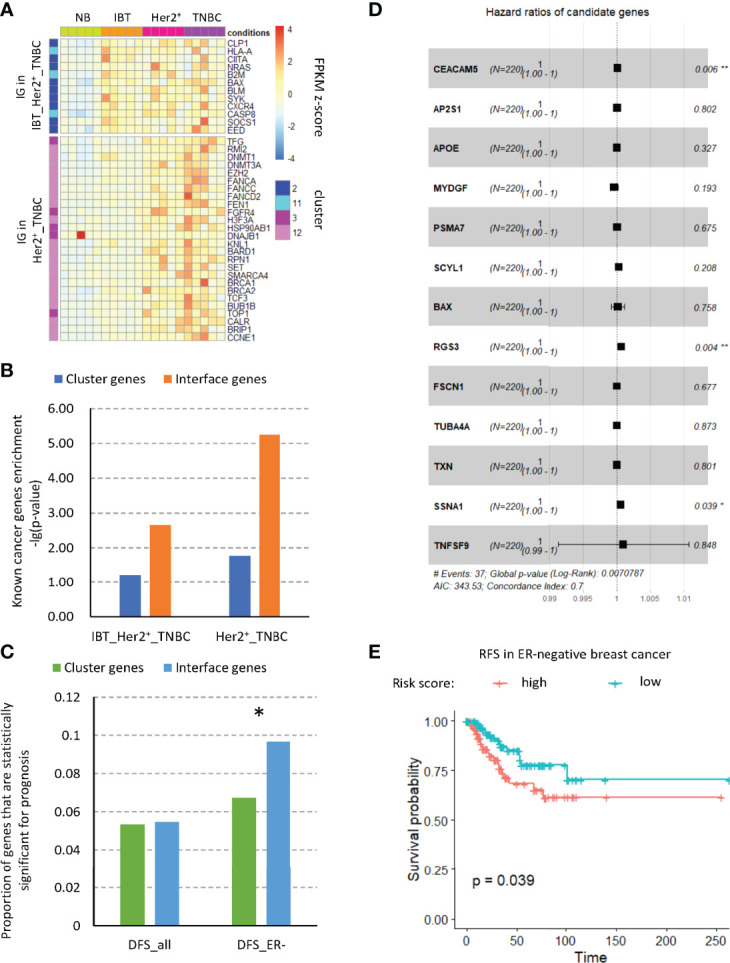
Interface genes were enriched in cancer-related genes. **(A)** The expression of known tumor-related genes in interface genes from IBT_Her2^+^_TNBC module and Her2^+^_TNBC module. **(B)** Enrichment of known tumor-related genes in different modules. **(C)** Proportion of genes with prognostic significance. Cluster, genes in clusters 2, 11, 3, and 12; IG, interface genes; DFS, disease-free survival; all, all breast cancer types; ER^−^, ER-negative breast cancer. **(D)** Forest plot of the hazard ratio (HR) for the association of risk factor gene expression with DFS. **(E)** Disease-free survival curves of high- and low-risk score groups based on multivariate Cox regression analysis. *, p-value < 0.05; “**”, p-value < 0.01.

A major goal of cancer research is to find those driver genes that contribute to tumor progression due to acquired mutations. For this reason, many methods and databases have been developed to analyze cancer data, such as COSMIC (34), DriverDB (35), and the Cancer Gene Census (CGC) (36). By integrating the CGC database and artificially screened genes, Repana et al. obtained a set of 711 known cancer genes, which included cancer suppressor genes and oncogenes and was experimentally verified (37). Therefore, we detected the expression of known cancer genes in the interface genes and counted the numbers of known cancer-related genes in the interface genes and in the different modules (**Figure 4A**). We found that the interface genes in the IBT_Her2^+^_TNBC module and the Her2^+^_TNBC module were significantly enriched for known cancer-related genes (**Figure 4B**). Among the interface genes in the IBT_Her2^+^_TNBC module, CXCR4 is a known cancer-related chemokine receptor gene. CXCR4 promotes breast cancer growth in three main ways: promoting angiogenesis, participating in the signal pathway of cell proliferation, and recruiting immune cells (38). The chemokine receptor CXCR4 not only plays a key role in tumorigenesis and cancer progression, but it is also an effective prognostic factor for breast cancer. Overexpression of CXCR4 can increase the risk of distant metastasis of breast cancer and reduce the overall survival rate and disease-free survival rate of patients (39). Among the interface genes in the Her2^+^_TNBC module, the known cancer-related genes are mostly DNA repair genes, such as BARD1, BRIP1, BRCA1, BRCA2, FANCA, FANCC, FANCD2, and FEN1. These interface genes should be enriched with key genes related to inflammation and cancer transformation.

Cancer driver genes have a key prognostic role in breast cancer and may serve as prognostic markers of breast cancer and provide treatment strategies for clinical treatment (40). Therefore, we tested the prognosis of all interface genes on DFS (disease-free survival) of different types of patients in the TCGA database. We found that, among the interface genes, the proportion of genes with significance prognosis of DFS in ER^−^ patients was significantly increased (**Figure 4C**). Therefore, we used single-factor Cox regression to detect the significance of all interface genes on the prognosis of RFS and selected those genes whose high expression would increase the risk of disease. By the multivariate Cox regression analysis of these genes, we established a disease occurrence prediction model consisting of nine risk factors (**Figure 4D**). The concordance index of this model is 0.7, which means that it has moderate predictive power. According to this model, we calculated the risk scores of ER^−^ patients in the TCGA database and divided the patients into high-risk groups and low-risk groups based on risk scores. According to the survival analysis results of DFS (**Figure 4E**), we found that the disease-free survival rate of the high-risk group was significantly reduced, which implied that this risk model might predict an increase in the incidence of cancer. In addition, to explore the related functions of risk factors, we extracted the related genes of these nine risk factors from the established modular protein interaction network (**Supplementary Figure3**). Through KEGG and GO functional enrichment analysis, we found that 5.53% of the functional items were related to the T cell receptor signaling pathway, which suggested the potential role of immune cells, especially T cells, for cancer (**Figure S3**).

### Treg cell immune infiltration increased in inflammation and ER^−^ breast cancer tissues

Because the interface genes in the IBT_Her2^+^_TNBC module were enriched in immune signaling pathways, we investigated the changes of the immune cells in different types of samples. On the basis of the gene expression signatures of 22 immune cell types, we used the CIBERSORT (22) deconvolution algorithm to infer the relative content of immune cell types in each sample.

**Figure 5:**
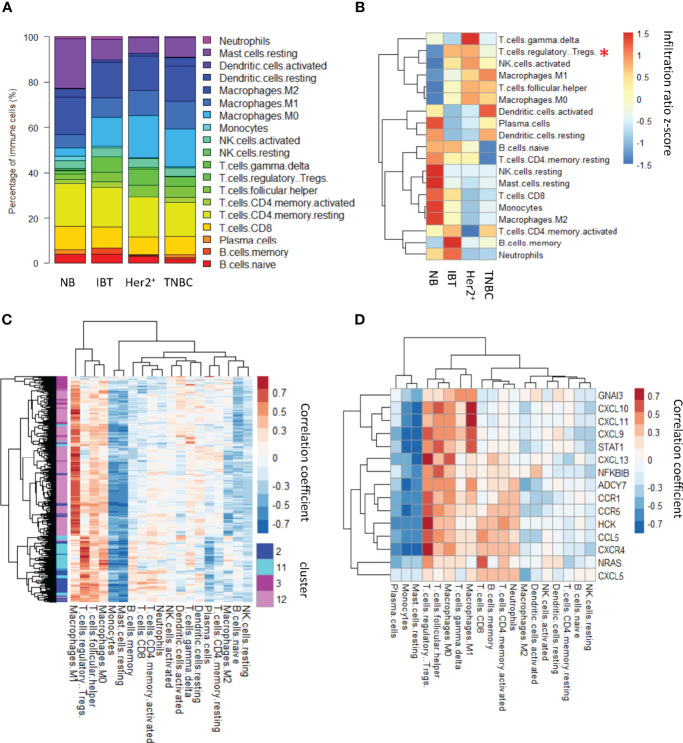
Treg cell immune infiltration was increased in inflammation and ER^−^ breast cancer. **(A)** Relative contents of immune cells between normal, inflammation, and cancer stages. **(B)** A heatmap showing average content of immune changes between normal, inflammation, and cancer stages. “*”, p-value < 0.05; “**”, p-value < 0.01 and “***”, p-value < 0.001. **(C)** The correlation between interface genes and immune cell infiltration. **(D)** The correlation between chemokine signaling pathway genes in interface genes and immune cell infiltration.

We calculated the average proportion of each type of immune cells in different sample types (**Figure 5A**) and found that regulatory T cells, activated NK cells, macrophages M1, follicular helper T cells, and macrophages M0 have a higher proportion both in inflammation breast tissues and ER^−^ breast cancer than normal tissues (**Figure 5B**). Among them, the ratios of regulatory T cells in IBT and ER^−^ breast cancer are statistically significant. Comparing the expression levels of interface genes with immune cell infiltration, it could be found that most of the interface genes from IBT_Her2^+^_TNBC are mainly related to Treg cell infiltration, whereas the interface genes from Her2^+^_TNBC are most related to the infiltration of macrophage M1 (**Figure 5C**). Because the interface genes from IBT_Her2^+^_TNBC enriched the genes of the chemokine signaling pathway, we tested the correlation between all the genes of the chemokine signaling pathway in the interface genes and immune cell infiltration. Most of these genes are positively correlated with Treg cells, indicating that the activation of chemokine signaling pathways may be related to Treg cell infiltration (**Figure 5D**).

Furthermore, recent studies showed that the erythroid cells played an important role in tumorigenesis (41, 42). To dissect the role of erythroid cells during the inflammatory-cancer transformation. We investigated the proportion of erythroid cell infiltration by CIBERSORT algorithm (22). The signature genes were retrieved from scRNA-seq for blood samples (24). In this study, the erythroid cells were further divided into seven groups including mature_RBCs, Transition_differentiating cells, ACVR2B_type, F cells, Reticulocytes, HEMGN_type, and NIX_type. We analyzed the infiltration of these seven groups in each sample type. Only four groups—mature_RBCs, Transition_differentiating cells, F cells, and NIX_type—were detected in our datasets. There was no significant difference in the infiltration of total erythroid cells among different stage (**Supplementary Figure 4A**). Among the different types of tissues, we observed that the contents of mature_RBC and F_cells were highest in the normal breast tissues. On the other hand, the contents of Transition_differentiating cells and NIC_type were highest in the TNBC (**Supplementary Figure 4B**). The opposite cell content changes suggested that erythroid cells might be involved in the inflammatory-cancer transformation and different subtypes of erythroid cells might play an important role in different stage of inflammatory-cancer transformation.

### Chemokines might be key regulators in inflammation-cancer transformation

Because the chemokine signaling pathway was enriched in the interface genes in the IBT_Her2^+^_TNBC module, we wondered whether it is universal changes in other inflammation-cancer transformation models. At present, some cancer types are thought to have evolved from inflammatory lesions, including HCC and CRC (12, 43). Although hepatitis B virus (HBV) and hepatitis C virus (HCV) are still the main risk factors for HCC, recently, non-alcoholic steatohepatitis (NASH) has become a frequent HCC risk factor in the West (44). Chronic inflammation of the intestinal mucosa may cause ulcerative colitis or Crohn’s disease and other inflammatory bowel disease (IBD) and further increase the risk of CRC in patients, which indicates that colon cancer is a typical inflammation-dependent cancer (45). The interface genes in the IBT_Her2^+^_TNBC module were enriched in IBD and hepatitis B signaling pathways, suggesting that inflammatory breast tissues might also contain similar factors and mechanisms that promote the inflammation-cancer transformation (**Figure 3B**).

**Figure 6:**
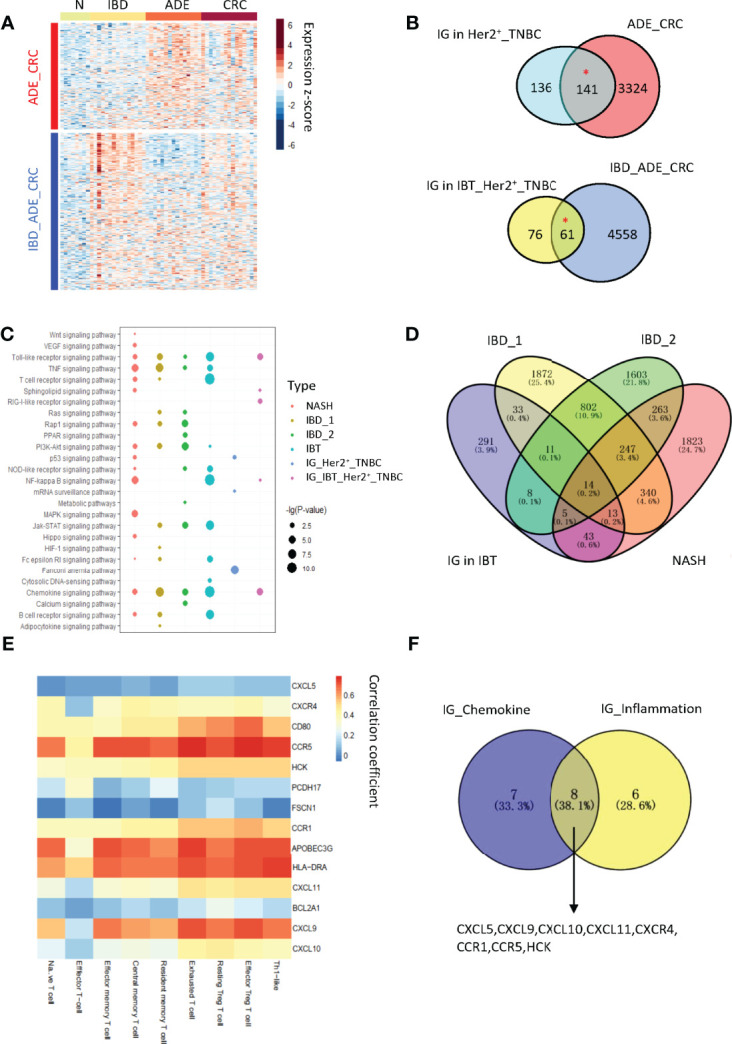
The expression of interface genes in other inflammatory diseases. **(A)** A heatmap of differentially expressed upregulated genes in the GSE4183 dataset. **(B)** The overlap between interface genes and differentially upregulated gene groups in IBD. “*”, p-value < 0.05. **(C)** Enrichment of signaling pathways in different inflammatory tissues. NASH (non-alcoholic steatohepatitis) and IBD (inflammatory bowel disease). IBT, inflammatory breast tissue; IG, interface genes. IBD_1, GSE4183; IBD_2, GSE83687; NASH, GSE162694; IG_IBT_Her2^+^_TNBC, interface genes in IBT_Her2^+^_TNBC module; IG_Her2^+^_TNBC, interface genes in Her2^+^_TNBC module. **(D)** Venn plot presents overlaps of interface genes and differentially expressed genes among NASH (non-alcoholic steatohepatitis) and IBD (Inflammatory Bowel Disease). IBT, inflammatory breast tissue; IG, interface genes. IBD_1, GSE4183; IBD_2, GSE83687; NASH, GSE162694. **(E)** The correlation between overlapped Genes and immune T cells infiltration in breast cancer samples. **(F)** Venn diagram of the interface genes from the chemokine signaling pathway and 14 overlapped genes. IG_Chemokine, interface genes involved in chemokine signaling pathway; IG_inflammation, interface genes that were highly expressed in other inflammations.

Therefore, we downloaded public datasets of inflammation and cancer samples from the Gene Expression Omnibus (GEO) database, including two IBD dataset (GSE4183 and GSE83687) and one liver dataset (GSE162694). Among them, the GSE4183 dataset contained samples of normal tissues, IBD tissues, adenocarcinomas, and CRC, which were similar to our samples. Therefore, we tested the differentially expressed upregulated genes in inflammatory tissues and cancer tissues, and we divided differentially upregulated genes into two groups according to the expression levels. The group of genes whose expression was obviously upregulated in inflammation and also tended to be upregulated in cancer was called the IBD_ADE_CRC group, and the other group of genes was more significantly upregulated in cancer, called the ADE_CRC group (**Figure 6A**). We compared the interface genes with different expression trends detected in breast samples and found that these interface genes had similar expression trends in IBD (**Figure 6B**).

By comparing with their own normal tissue samples, the differentially expressed genes of inflammation samples from other IBD and NASH datasets were also identified. We tested the enrichment of signaling pathways in upregulated genes from different inflammatory diseases and found that the Toll-like signaling pathway and chemokine signaling pathway enriched in interface genes in the IBT_Her2^+^_TNBC module were significantly activated in NASH, IBD, and IBT samples (**Figure 6C**). To find common genes in inflammatory cancer transformation, we explored the overlap of interface genes and upregulated genes in NASH and IBD (**Figure 6D**). In the end, we found that 14 interface genes were consistently upregulated in NASH, IBD, and IBT samples. We tested the correlation between the expression of these 14 overlapped genes in breast cancer and immune T cell characteristics, and we found that more than half of the genes were positively correlated with Treg cells infiltration (**Figure 6E**). In addition, we found that eight of the 15 interface genes from the chemokine signaling pathway were also upregulated in the three types of inflammation samples, suggesting their potential role in inflammatory cancer transformation (**Figure 6F**). For example, CXCL9, CXLC10, and CXCL11/CXCR3 has two main functions: it activates the immune response through the paracrine pathway and promotes the proliferation and metastasis of cancer cells through the autocrine pathway (46). As an autocrine signal, CXCL9, CXCL10, or CXCL11 activates the chemokines produced by the tumor through CXCR3A on cancer cells and further recruits immune cells to create an immune microenvironment that promotes tumors. In addition, we found that APOBEC3G was highly expressed in all inflammation tissues and was positively correlated with Treg cell immune infiltration in breast tumors (**Figure 6E**). APOBEC3 family members are cytidine deaminases that can increase the probability of DNA mutations, indicating that the high expression of APOBGE3C in inflammatory lesions may be one of the reasons that inflammation increases the risk of cancer.

## Discussion

It has been confirmed that chronic inflammation is an important cause of many malignant tumors, but its effect on the occurrence and development of breast cancer is still poorly understood (47). A variety of chronic inflammatory factors have been found to increase the risk of breast cancer, suggesting that chronic inflammation may play a role in the initiation and development of breast cancer (13). Here, we compared the transcriptional expression profiles of normal tissues, inflammatory lesions of the breast, benign breast tumors, and malignant breast tumors. We found that inflammatory lesions of the breast shared many similarities with ER^−^ malignant tumors, such as low ER expression levels, and similar immune signaling pathway activation. Previous studies have found that the degree of immune infiltration is positively correlated with ER^−^, including lymphocyte infiltration, plasma cell infiltration, macrophage infiltration, and other inflammatory cell infiltration (48). Our data showed that macrophages M1 and macrophages M0 had a higher proportion both in inflammation and ER^−^ cancer than normal tissues. All these make us state that there is a closer connection between inflammatory breast tissues.

Because of the similarities between inflammatory lesions of the breast and ER^−^ breast cancer, we focused our attention on the relationship between them. On the basis of the gene expression trend in the sample, we identified the IBT_Her2^+^_TNBC module and Her2^+^_TNBC module. By constructing the protein–protein interaction network between the IBT_Her2^+^_TNBC module and the Her2^+^_TNBC module, we found the “inflammation cancer interface” and identified 133 and 278 interface genes for each module, respectively. Interface genes had a significant enrichment effect on cancer-related genes, suggesting that interface genes might be involved in the process of cancer occurrence and tumor formation. KEGG signaling pathway analysis showed that interface genes were enriched in immune-related signaling pathways, for example, chemokine signal pathway, and NF-κB signal pathway. Chronic activation of NF-κB can lead to the development of various autoimmune, inflammation-related diseases and solid tumors (49). Chemokines are a class of small secretory molecules that regulate the migration of immune cells by binding to receptors. In IBD and non-alcoholic steatohepatitis (NASH), two types of inflammatory diseases that may cause cancer, we found that all chemokines and chemokine receptors in interface genes had higher expression compared with normal tissues. In many cancer types, chemokines can regulate the composition of leukocyte infiltration (50). There is evidence that CCR5, one of interface genes in IBT_Her2^+^_TNBC, interacts with CCl5 to promote tumorigenesis at the beginning of cancer (51). Among them, CXCL9, CXCL10, and CXCL11 have been reported to promote cancer cell proliferation by combining with CXCR3A (46). The interface gene set was highly expressed and enriched in IBD and NASH, indicating the promotion of the interface gene in the transformation of inflammatory cancer.

In addition, it has also been seen that Epstein–Barr virus (EBV) infection, viral myocarditis, and pathogenic *Escherichia coli* infection were enriched in interface genes. Now, breast tissue is considered to have this specific microbiome rather than a sterile environment. The microbiome in breast cancer adjacent tissues is significantly different from normal breast tissue, suggesting its possible role in breast cancer (52). Studies have found that the genera *Fusobacterium* is more abundant in breast cancer adjacent tissues than in normal breast tissues, and it may cause cancer by releasing factors and providing a pro-inflammatory environment. In addition, early studies have shown that human papillomavirus (HPV) exhibits an association with breast cancer, which may promote the progression of inflammatory response in breast tissue and thus benefit the process of breast cancer (53, 54). Lymphotropic viruses, such as EBV, can continuously activate JAK-STAT and NF-κB pathways (49). The long-term STAT and NF-κB activation of these viruses leads to the induction of chronic inflammation, which can support the persistence of these viruses and promote virus-mediated cancer (49). This suggests that viruses and microbiomes may become a source of inflammation in tissues and increase the risk of cancer.

In summary, we found the similarity between inflammatory lesions of the breast and ER^−^ breast cancer and analyzed their transcriptome. Through comprehensive protein network analysis, we identified the interface genes and signaling pathways that have the potential to promote inflammatory cancer transformation. We speculate that, in inflammation lesions, which might be caused by changes of the virus and microbial populations, constantly highly expressed APOBGE3C would increase the probability of DNA mutations and increase the risk of cancer; on the other hand, inflammation recruits immune cells, such as Treg cells, and Treg cells secreted chemokines and activated chemokine signaling pathways to promote cancer cells proliferation. These interface genes can be used as a risk factor to provide a certain basis for the clinical early detection and treatment of breast cancer. The correlation analysis between inflammatory lesions of the breast and ER^−^ breast cancer provides a basis for the continued analysis of inflammatory cancer transformation analysis.

## Data availability statement

The data presented in the study are deposited in the NCBI repository, accession number PRJNA855324.

## Ethics statement

The studies involving human participants were reviewed and approved by institution’s ethics committee, Fudan University Shanghai Cancer Center Institutional Review Board. The patients/participants provided their written informed consent to participate in this study.

## Author contributions

SL designed and supervised the study and wrote the manuscripts. ZC analyzed and interpreted the RNA-seq data and wrote the manuscripts. YZ and GL collected and prepared the patient samples for RNA-seq. JT wrote the manuscript. JF assisted the RNA-seq. LZ provided helpful suggestions. All authors have reviewed the manuscript and approved the final version.

## Funding

This work was supported by the National Key Research and Development Program of China (Stem Cell and Translational Research 2020YFA0112300); National Natural Science Foundation of China (81930075, 81772799); “Ten Thousand Plan” - National High-Level Talents Special Support Plan WR-YK5202101; Program for Outstanding Leading Talents in Shanghai; Program for Outstanding Medical Academic Leader in Shanghai (2019LJ04); Program of Shanghai Academic/Technology Research Leader 20XD1400700; the innovative research team of high-level local university in Shanghai; the Fudan University Research Foundation (IDH 1340042); and the Research Foundation of the Fudan University Shanghai Cancer Center (YJRC1603).

## Acknowledgments

The results here are partly based on data generated by the TCGA research network: https://www.cancer.gov/tcga.

## Conflict of interest

Author FJ was employed by Singleron Biotechnologies.

The remaining authors declare that the research was conducted in the absence of any commercial or financial relationships that could be construed as a potential conflict of interest.

## Publisher’s note

All claims expressed in this article are solely those of the authors and do not necessarily represent those of their affiliated organizations, or those of the publisher, the editors and the reviewers. Any product that may be evaluated in this article, or claim that may be made by its manufacturer, is not guaranteed or endorsed by the publisher.

## Supplementary material

Supplementary Figure 1 The performance characteristics of special genes in IBT. **(A)** Breast cancer types predicted by the expression of PAM50 characteristic genes in IBT. Expression of ESR1 **(B)**, PGR **(C)** and ERBB2 **(D)** among different breast tissues. “ns”, p-value > 0.05; “**”, p-value < 0.01.

Supplementary Figure 2 The expression clusters of differentially expressed genes in IBT and ER^−^ breast cancer samples (Her2^+^ and TNBC). The main enriched functional items are listed on the right. The heatmap on the left showed the average expression value of the cluster in each type of sample and trend graph on the right showed normalized gene expression and the cluster expression values.

Supplementary Figure 3 NP network and function enrichment of risk factors. **(A)** NP network of risk factors. The color of the node indicated genes from different groups, and the color of the edge indicated the correlation coefficient of expression between gene nodes. **(B)** Proportion of enriched KEGG and GO terms of genes in the NP network of risk factors

Supplementary Figure 4 erythroid cell infiltration in inflammation and ER^−^ breast cancer. **(A)** The absolute score of erythroid cells among different breast tissues. “ns”, p-value > 0.05. Absolute score reflected the absolute proportion of blood cells in a mixture. **(B)** A heatmap showed the average content of different erythroid cells among normal, inflammation, and cancer stages.
