# Supplementary figures and images for: The potential effects and mechanisms of breast inflammatory lesions on the occurrence and development of breast cancer
Source: Front Oncol. 2022 Aug 5;12:932743. doi: 10.3389/fonc.2022.932743 (PMC9389363; doi:10.3389/fonc.2022.932743)

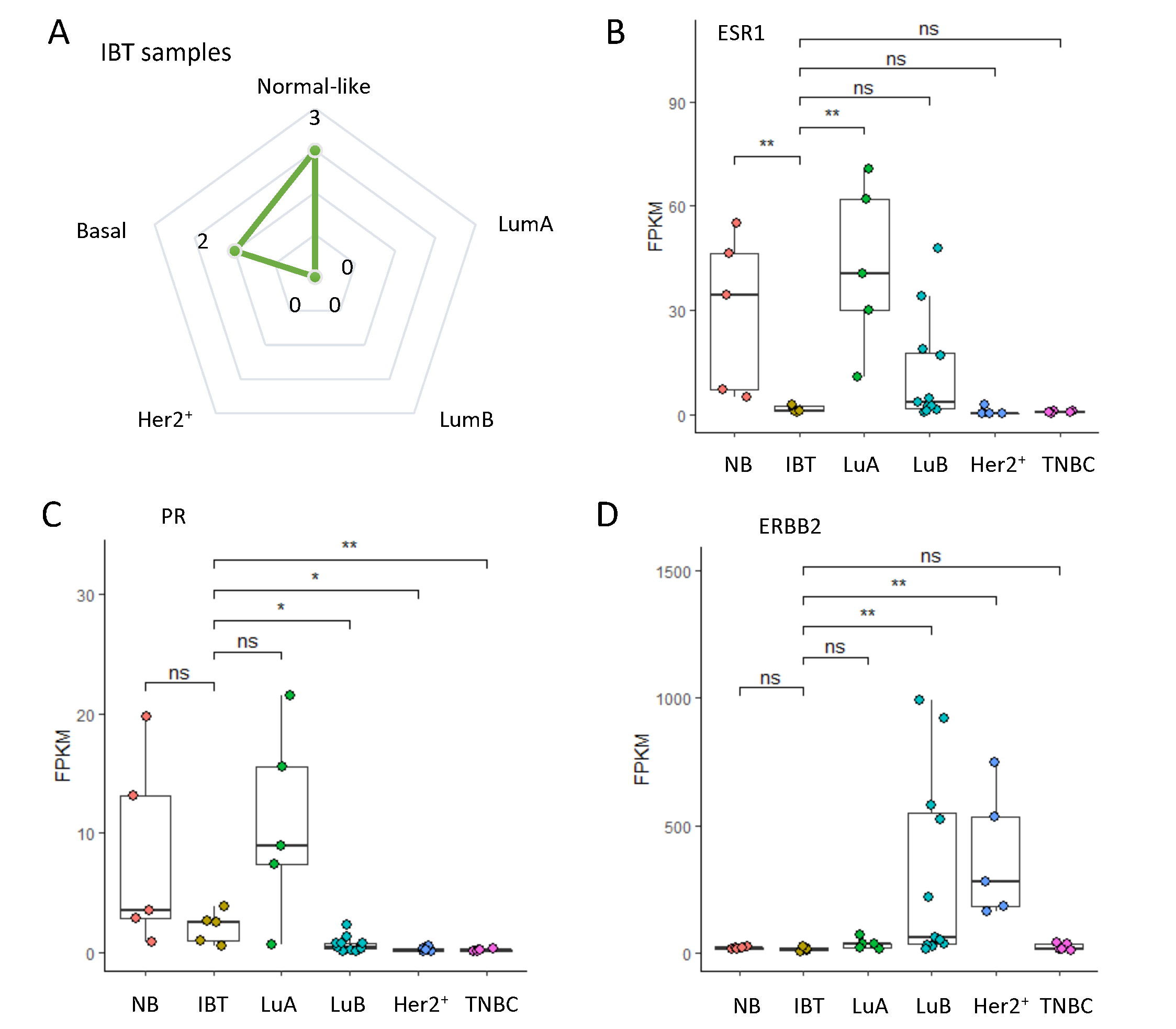

Supplement: Supplementary Figure 1 — The performance characteristics of special genes in IBT. (A) Breast cancer types predicted by the expression of PAM50 characteristic genes in IBT. Expression of ESR1 (B), PGR (C) and ERBB2 (D) among different breast tissues. “ns”, p-value > 0.05; “**”, p-value < 0.01. [file Image_1.tiff]

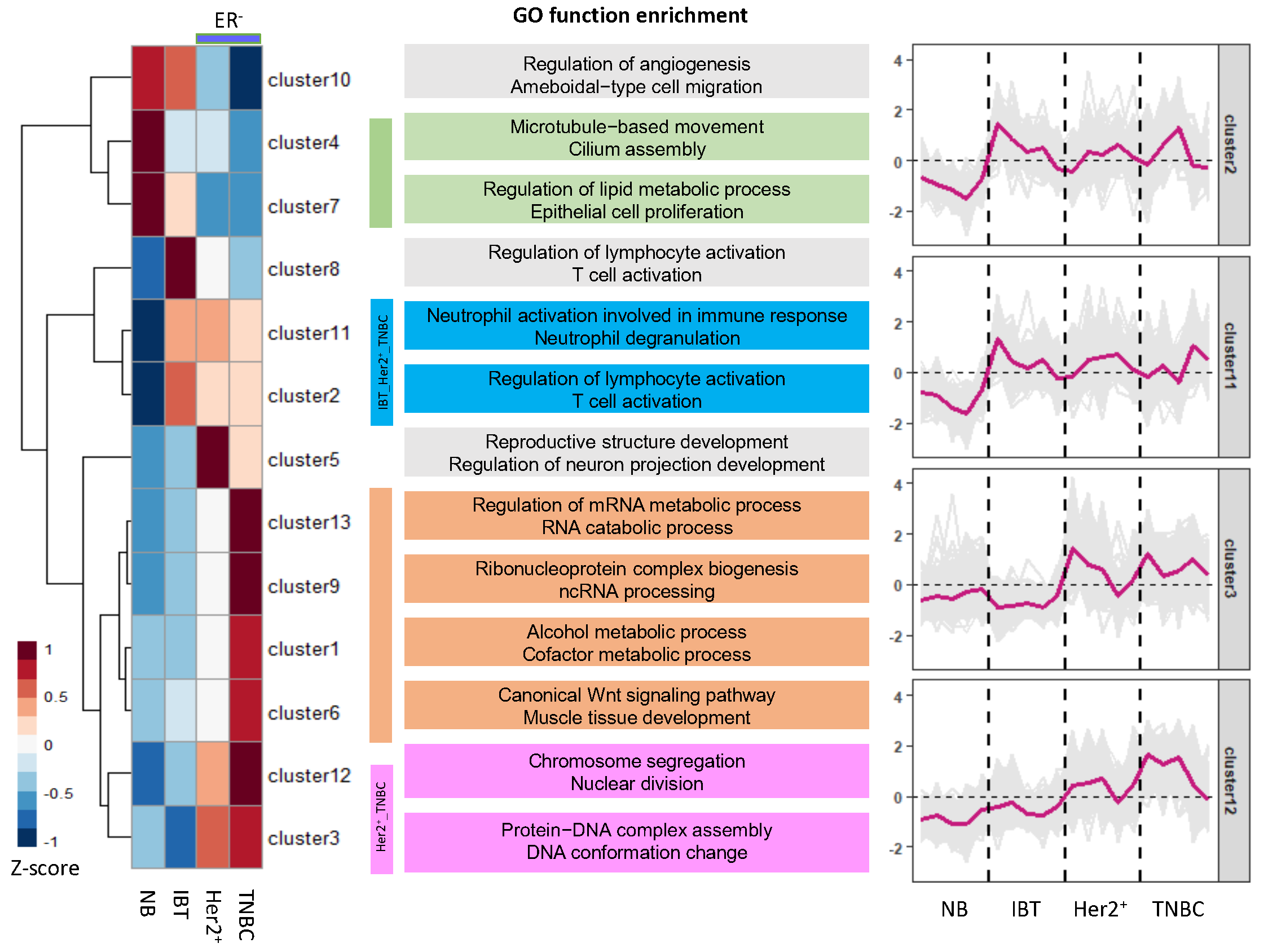

Supplement: Supplementary Figure 2 — The expression clusters of differentially expressed genes in IBT and ER− breast cancer samples (Her2+ and TNBC). The main enriched functional items are listed on the right. The heatmap on the left showed the average expression value of the cluster in each type of sample and trend graph on the right showed normalized gene expression and the cluster expression values. [file Image_2.tiff]

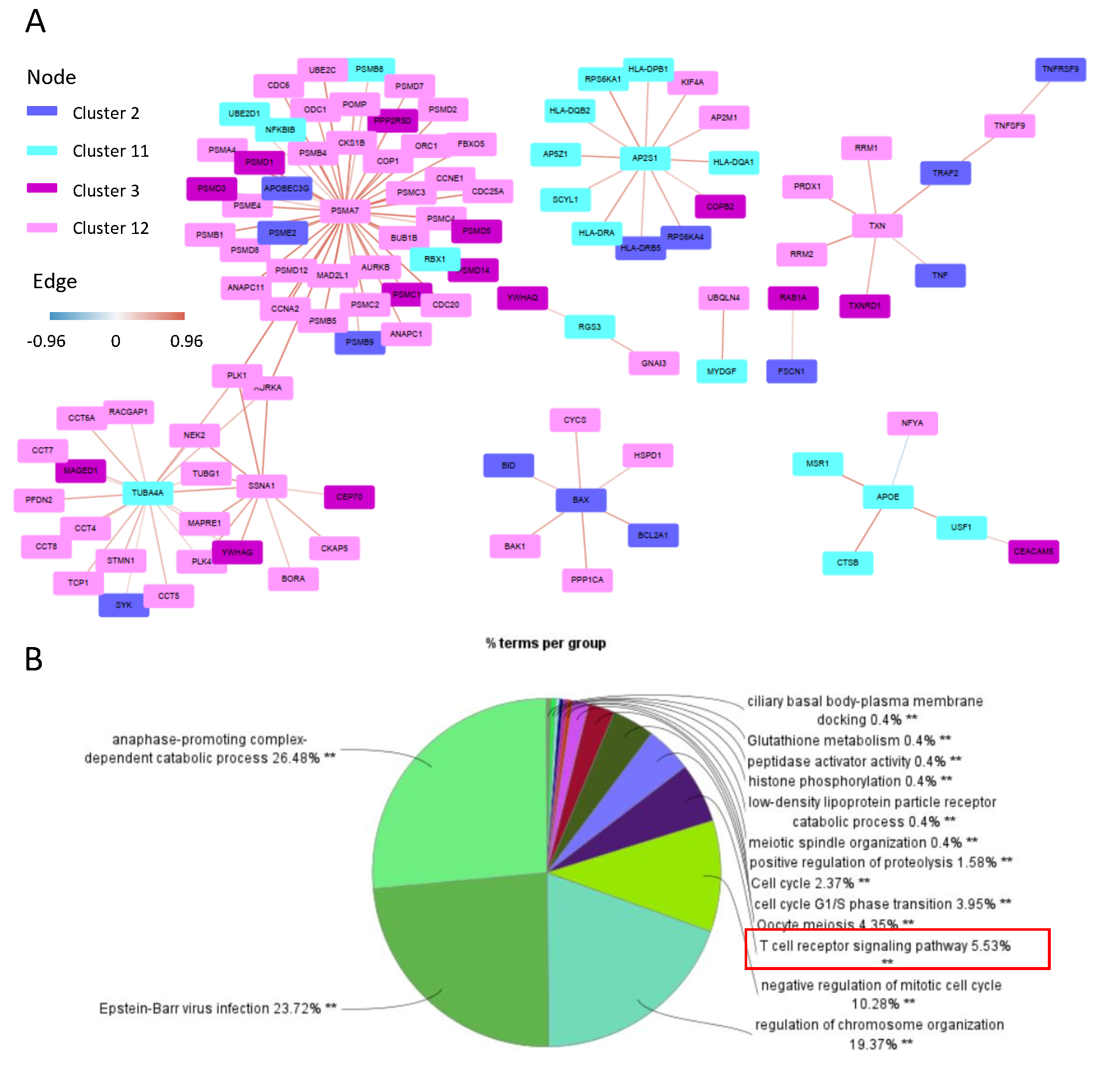

Supplement: Supplementary Figure 3 — NP network and function enrichment of risk factors. (A) NP network of risk factors. The color of the node indicated genes from different groups, and the color of the edge indicated the correlation coefficient of expression between gene nodes. (B) Proportion of enriched KEGG and GO terms of genes in the NP network of risk factors [file Image_3.tiff]

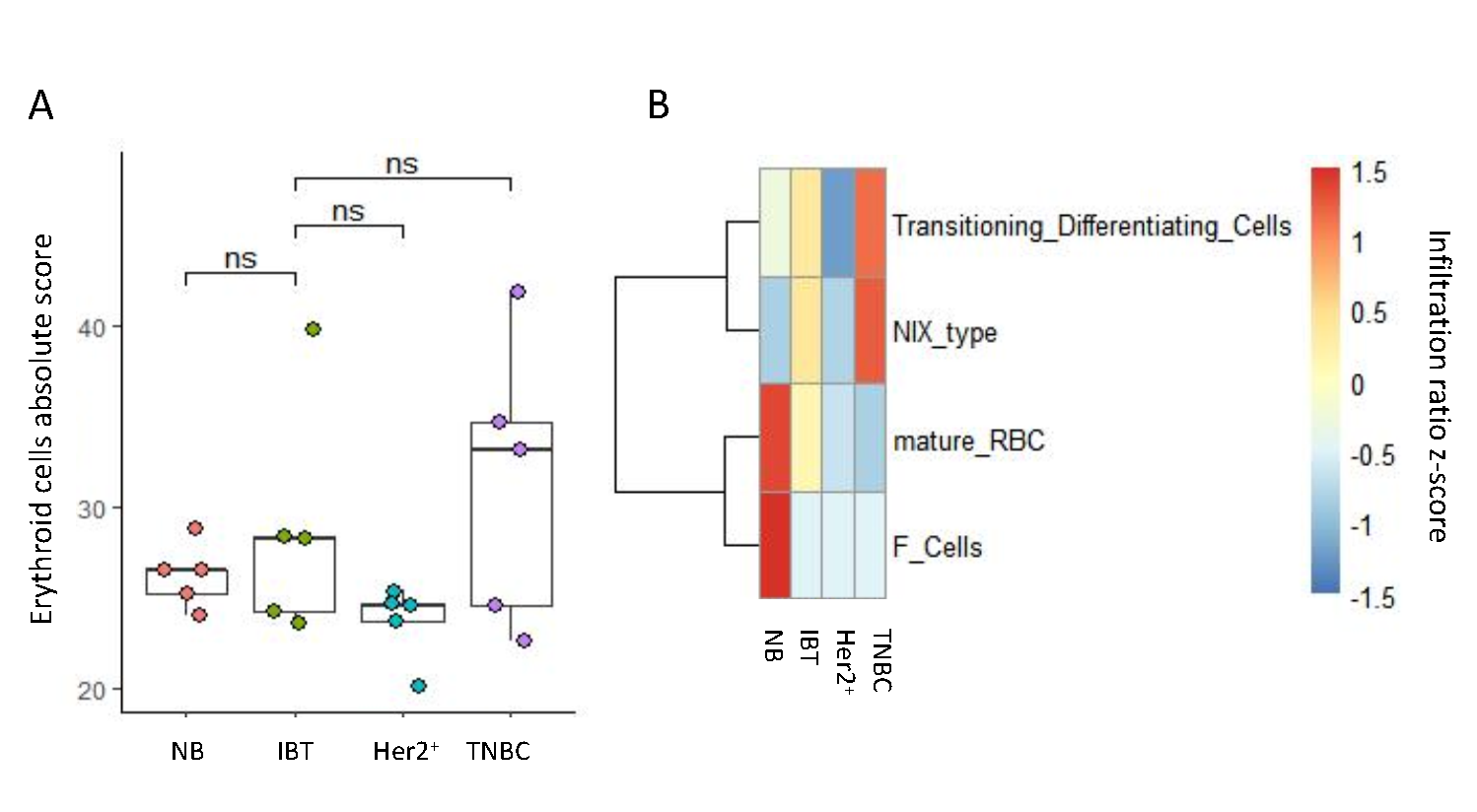

Supplement: Supplementary Figure 4 — erythroid cell infiltration in inflammation and ER− breast cancer. (A) The absolute score of erythroid cells among different breast tissues. “ns”, p-value > 0.05. Absolute score reflected the absolute proportion of blood cells in a mixture. (B) A heatmap showed the average content of different erythroid cells among normal, inflammation, and cancer stages. [file Image_4.tiff]
